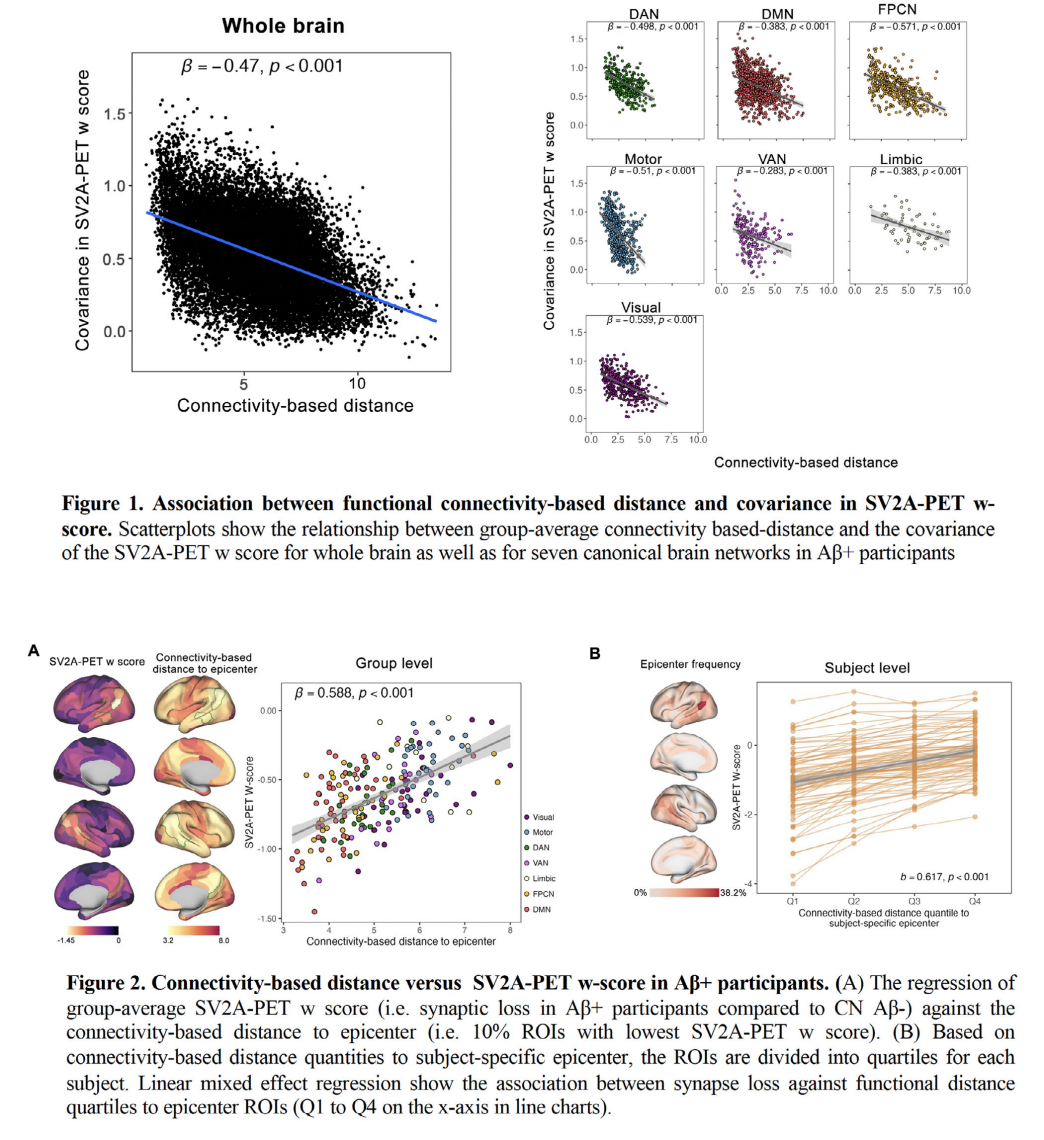# Synaptic loss pattern is associated with functional connectivity in Alzheimer’s disease

**DOI:** 10.1002/alz.085547

**Published:** 2025-01-09

**Authors:** Ying Luan, Weiyi Wang, Qi Huang, Yihui Guan, Binyin Li, Michael Schöll, Nicolai Franzmeier, Fang Xie

**Affiliations:** ^1^ Huashan Hospital, Fudan University, Shanghai China; ^2^ Zhongda Hospital, School of Medicine, Southeast University, Nanjing China; ^3^ Huashan Hospital, Fudan University, Shanghai, Shanghai China; ^4^ Ruijin Hospital affiliated to Shanghai Jiaotong University School of Medicine, Shanghai China; ^5^ Institute of Neuroscience and Physiology, Sahlgrenska Academy, University of Gothenburg, Gothenburg Sweden; ^6^ Institute for Stroke and Dementia Research (ISD), University Hospital, LMU, Munich, Bayern Germany

## Abstract

**Background:**

Synaptic loss is identified as a strong correlate of cognitive impairment in Alzheimer’s disease (AD). Pathological tau can induce direct toxicity to synapse and spread trans‐synaptically across connected neurons. Recent neuroimaging evidence revealed that tau pathology propagates along interconnected brain regions throughout macroscale brain networks. However, whether synaptic loss propagates in AD follows a similar vein is unclear.

**Method:**

Seventy‐six amyloid‐positive (Aβ+) subjects across AD spectrum and 48 cognitively normal (CN) Aβ‐ controls characterized by cross‐sectional [^18^F]florbetapir amyloid‐PET were included in the current study. The density of synaptic vesicle glycoprotein 2A (SV2A) was measured by [^18^F]SynVesT‐1 PET. A normative template of functional connectome distance across 200 neocortical regions of interest (ROIs) was generated using resting‐state fMRI data from 1000 subjects of the Human Connectome Project. We assessed the association between synaptic loss and functional connectivity by correlating the cross‐subject inter‐regional covariance with the normative functional connectivity template. In a next step, we tested whether the functional distance to synaptic loss epicenter (i.e., top 10% ROIs with greatest synaptic loss) is associated with synaptic loss in connected regions at both group‐ and subject‐level.

**Result:**

Among Aβ+ subjects, inter‐regional covariance of SV2A‐PET‐assessed synaptic loss was associated with inter‐regional functional connectivity (Figure 1), suggesting that higher functional connectivity may facilitate the propagation of synaptic loss. At both group‐ and subject‐level, we found that shorter functional distance to synaptic loss epicenters is associated with greater levels of synaptic loss in connected regions (Figure 2).

**Conclusion:**

We found that inter‐regional functional connectivity is associated with greater synaptic loss in AD, suggesting that synaptic loss may systematically propagate along brain connections.